# Electrochemical Determination of Low Molecular Mass Thiols Content in Potatoes (*Solanum tuberosum*) Cultivated in the Presence of Various Sulphur Forms and Infected by Late Blight (*Phytophora infestans*)

**DOI:** 10.3390/s8053165

**Published:** 2008-05-15

**Authors:** Pavel Ryant, Eva Dolezelova, Ivo Fabrik, Jiri Baloun, Vojtech Adam, Petr Babula, Rene Kizek

**Affiliations:** 1 Depatment of Agrochemistry, Soil Science, Microbiology and Plant Nutrition, Mendel University of Agriculture and Forestry, Zemedelska 1, CZ-613 00 Brno, Czech Republic; 2 Department of Chemistry and Biochemistry, Mendel University of Agriculture and Forestry, Zemedelska 1, CZ-613 00 Brno, Czech Republic; 3 Department of Animal Nutrition and Forage Production, Faculty of Agronomy, Mendel University of Agriculture and Forestry, Zemedelska 1, CZ-613 00 Brno, Czech Republic; 4 Deparment of Natural Drugs, Faculty of Pharmacy, University of Veterinary and Pharmaceutical Sciences, Palackeho 1-3, CZ-612 42 Brno, Czech Republic

**Keywords:** Thiols, Glutathione, Plant, Potato (*Solanum tuberosum*), Heavy metal, Cadmium, Late blight (*Phytophora infestans*), Electrochemical methods, Differential pulse voltammetry Brdicka reaction

## Abstract

In the present paper potato plants were cultivated in the presence of ammonium sulphate or elemental sulphur supplementation into the soil to reveal the effects of different sulphur forms on content of nitrogen, phosphorus, potassium, calcium, magnesium and sulphur, and yield of tubers. During the investigation of the influence of different sulphur forms on yield of potato tubers we did not observe significant changes. Average weight of tubers of control plants per one experimental pot was 355 g. Application of sulphur in both forms resulted in moderate potato tubers weight reduction per one experimental pot compared to control group; average value ranged from 320 to 350 g per one experimental pot. Further we treated the plants with two different supplementation of sulphur with cadmium(II) ions (4 mg of cadmium(II) acetate per kilogram of the soil). The significantly lowest cadmium content (p < 0.05) was determined in tissues of plants treated with the highest dosage of elemental sulphur (0.64 mg Cd/kg) compared to control plants (0.82 mg Cd/kg). We also aimed our attention on the cadmium content in proteins, lipids or soluble carbohydrates and ash. Application of sulphate as well as elemental sulphur resulted in significant cadmium content reduction in lipid fraction compared to control plants. In addition to this we quantified content of low molecular mass thiols in potatoes tissues. To determine the thiols content we employed differential pulse voltammetry Brdicka reaction. After twelve days of the treatment enhancing of thiols level was observed in all experimental groups regardless to applied sulphur form and its concentration. Finally we evaluated the effect of sulphur supplementation on *Phytophora infestans* infection of potato plants.

## 1. Introduction

The sulphur oxides emissions into atmosphere were dramatically reduced in central Europe at the end of the last century mainly due to thermal power station modernization, reduction of fossil fuels combustion etc. Nowadays the level of sulphur oxides emissions is reduced for more than 70 % compared to emissions in 1980 [1-5]. Sulphur itself is one of the most important macroelements. As element it is a part of many important proteins, enzymes, secondary metabolites participating in number of plant biochemical pathways including a plant stress response. Sulphur, especially its form and amount, plays important role in processes of heavy metals ions uptake and subsequent distribution and deposition in different plant organs. This point is very important for certain plant species cultivation. If thiols, compounds with free –SH group, are able to prevent plant from deposition of higher heavy metals ions concentrations into their consumption parts (such as fruits), they enable us to utilize them for safety (heavy metals free) food production. The reduction of anthropogenic sulphur deposition resulted in progressive sulphur deficiency in plant mineral nutrition [6-13]. Therefore the sulphur or compounds containing sulphur (organic as well as inorganic forms) are supplemented most frequently by two different ways; the first one is the foliar application where sulphur is introduced directly onto aerial plant parts, especially leaves from where it is rapidly absorbed and integrated directly into plant metabolism. The second way is the application of sulphur or its compounds directly into soil. Plants obtain sulphur from soil by the use of many mechanisms, which can effectively regulate the rate of assimilation [14-23]. The fertilizers containing sulphur in sulphate or elemental form are commonly used. Sulphate form is highly soluble and mobile in soil profile; in contrary elemental form is water-insoluble and thus can not be taken up by plants. This form must be metabolized by soil microorganisms, which results in their oxidation to sulphates [24].

**Figure 1.**
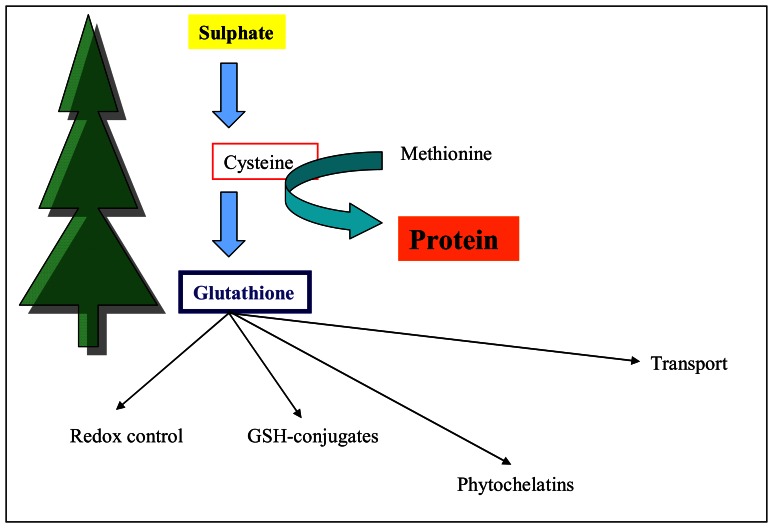
Possible scheme of metabolizing of sulphate in a plant.

The reduction of sulphates to S^2-^ results in their incorporation into cysteine and methionine. These sulphur amino acids are essential for biosynthesis of other biologically important thiols such as the most abundant non-protein thiol called glutathione and phytochelatins playing a key role in heavy metals detoxification processes (Fig. 1). In addition there are synthesized cysteine-rich secondary metabolites such as glucosinolates, which are typical for *Brassicales* plants. Other secondary metabolites, especially non-protein sulphur amino acids, were found in the most of *Allium* species [14-23].

It was shown that plants sufficiently supplemented by sulphur are able to excrete hydrogen sulphide out of the leaves surface to protect themselves against pathogens able to invade the plant [25,26]. Exact mechanism of the action as well as of way of synthesis and excretion of sulphide is still not clear. The most currently accepted opinion is that the synthesis is connected with biosynthesis of low molecular mass stress thiols. These features can be very important in attacking the potato plants (*Solanum tuberosum*, Solanaceae) by the most dangerous pathogen called late blight (*Phytophora infestans*, Pythiaceae). Late blight is still difficult to control by common methods and, thus, causes economically considerable losses. The etiological agent is the water mold *Phytophtora infestans* (Mont.) de Bary (*Pythiaceae*). The critical period occurs when the temperature ranges from 10 to 24 °C and the relative humidity is higher than 75 % for consecutive 48 hours. Under these conditions the late blight infection develops very quickly, especially on the surface of the damp leaves, often despite of recommended fungicides application.

This work was aimed on the investigation of sulphur supplementation on thiols content in potato plants infected or non-infected by late blight, or treated with cadmium(II) ions. To determine level of low molecular mass thiols differential pulse voltammetry Brdicka reaction was used.

## 2. Material and Methods

### 2.1 Plant material

Potato cultivar Marabel (*Solanum tuberosum* cv. Marabel) belongs to the early potato cultivar that develops low or medium-high semi-erect clusters of the transient type. Starting herb growth is medium-fast. Stems are thin to medium-thick; leaves are medium to large-sized, leaflet of medium size, medium wide, with weak to medium sinuate margins. The flowers are small to medium-sized, corolla is white. The flowers frequency is very low to low. This cultivar demonstrates very rapid growth of medium-sized tubers with lower starch content. This cultivar is resistant to viral diseases and common scab.

### 2.2 Experimental scheme of pot experiment

Ammonium sulphate and elemental sulphur were purchased from Lachema (Czech Republic). They were introduced into soil before own outplanting (April). At all experimental groups the nitrogen level was balanced by the ammonium sulphate. One potato tuber of cultivars Impala or Mabel was potted into one experimental pot. During vegetation experimental plants were watered by demineralised water and treated by the preparation Karate 2.5 WG against aphides. Sulphur foliar application was carried out at the beginning of June.

Own annual vegetative pot experiment was carried out in the open cultivation hall. The hall is placed in the area of Mendel University of Agriculture and Forestry, Brno - Cerna Pole, Czech Republic. Temperature was measured daily by weather station placed nearby the hall. Geographical latitude is 49°13′01″, terrestrial longitude 16°36′50″ and elevation above sea-level 250 m.

Ammonium sulphate as well as elemental sulphur was introduced directly to soil according to Table 1. Each experimental group included four repetitions. Plants were cultivated in medium-heavy alkaline soil (10 kg soil·pot^-1^) with the determined value of exchangeable pH (pH/CaCl_2_ 7.55) and nutrients content according Melich III (P 218 mg/kg soil; K 462 mg/kg soil; Ca 4718 mg/kg soil; Mg 346 mg/kg soil). For our experiments the soil was taken from plough layer of the land in University agriculture enterprise in žabčice, Czech Republic. This soil was typologically characterized as fluvial gley soil (FM_G_). Its granularity comes to the medium-heavy soil with humus content 2.44 %. Carbonates content is lower than 0.5 %. Aerial plant parts were harvested at 10^th^ July, which means at 76^th^ day of the cultivation. The harvesting of subterranean parts – tubers – followed at 13^th^ July (79th day of the cultivation). At the same time the soil was sampled. In plant mass the contents of P, K, Ca and Mg were determined after mineralization by sulphuric acid and hydrogen peroxide. The determination of nutrients (P, K, Ca and Mg) in soil samples was carried out according to Melich III. In addition the yield of tubers and herb, and total sulphur content in plants and soil were also quantified.

#### 2.2.1 Preparation of *Phytophora infestans* inoculum and evaluation of *Phytophora* infection

Foliar application of *Phytophtora infestans* inoculum was carried out in the stage of stand closing at the end of June. *Phytophtora infestans* was obtained from the isolate O1/6 and O1/3 supplied by Czech University of Life Sciences Prague, Czech Republic. Sporangia were washed away by the sterile distilled water. After spores releasing the suspension was incubated for 2 hours at 7 °C. Then the suspension was filtered and standardized to the inoculum density 2·10^3^ spores·ml^-1^. The rate of infestation of individual plants was evaluated at the 2^nd^ July 2004 (14 days after inoculation). The evaluation was based on the leaves spots percent occurrence, which is obviously closely related with the infection by this phyto-pathogen. These spots are yellowish to bright green at first. In our experiment only necrotic spots were evaluated.

#### 2.2.2 Cultivation of plants in soil supplemented by cadmium(II) ions

Cultivation of potato tubers was carried out in soil with natural cadmium content 0.182 mg Cd·kg^-1^. The soil was further supplemented by 4 mg of cadmium(II) acetate per kilogram of soil. Plants were cultivated under the same conditions as it is described in section 2.2.1. The control plants were those treated with cadmium(II) ions but without supplementation of sulphur.

### 2.3 Preparation of samples

Plants were washed with 1 M EDTA in 0.2 M phosphate buffer (pH 7.0) and centrifuged for 5 min at 3000 × *g* (Eppendorf 5402, USA). Plants (about 20 mg) washed of their cultivation media were frozen three times in liquid nitrogen to disrupt the cells and then transferred to a test tube. The frozen samples were homogenised by shaking on a Vortex–2 Genie for 5 min at 4 °C (Scientific Industries, USA) and sonicated using a Bandelin Sonopuls HD 2070 for 10 s at 7 W (Germany). The homogenate was centrifuged (14 000 × g) for 15 min at 4°C using a Universal 32 R centrifuge (Hettich-Zentrifugen, Germany). The supernatant was filtered through a 0.45 μm Nylon filter discs (Millipore, Billerica, Mass., USA) prior to electrochemical analysis using stationary electrochemical system.

### 2.4 Determination of nutrients according to Mehlich III

The contents of N, P, K, Ca and Mg were determined in the soil extracted by solution prepared as follows: one litre of the solution: 20 g ammonium nitrate, 4 ml ammonium fluoride-EDTA solution (per 500 ml: 69.45 g ammonium fluoride and 36.75 g EDTA was mixed at 50 °C), 11.5 ml nitric acid and 11.5 ml acetic acid. Briefly, soil (10 g, fine-grained sol) was extracted with 100 ml of the extraction solution under shaking for 10 min. The suspension obtained was filtered through paper filter. The filtrate was analysed.

The spectral determination of phosphorus was carried out on apparatus UNICAM 8625 (Unicam, Cambridge, Great Britain). The content of receivable calcium and magnesium was determined by using of atomic absorption spectrophotometer PU 9200X (Philips, Netherlands) in acetylene-air flame with deuterium background correction. Atomic emission spectrometer AAS 30 (Carl Zeiss Jena, Germany) was utilized for the determination of receivable potassium content.

### 2.5 pH of the soil

The value of pH was determined by pH metre MS 22 (Laboratorní přístroje Praque, Czech Republic) measuring hydrogen ions activity in the soil macerate with addition 0.01 M CaCl_2_.

### 2.6 Sulphur content in plants

Plant tissues were dissolved with the mixture of H_2_O_2_ and HNO_3_ in closed microwave system (MILESTONE ML 1200 MEGA, Bergamo, Italy). After this step, the sample was analysed utilizing optic emission spectrometry with inductively coupled argon plasma (ICP-OES) using the instrument JY-24 (JOBEN-YVON, France).

### 2.7 Determination of cadmium content in plants

Cadmium content was determined by flame atomic absorption spectrophotometer with deuterium background correction using apparatus Philips PU 9200X (Philips, Netherlands) after plant tissues dry-way mineralization.

### 2.8 Electrochemical measurement – Differential pulse voltammetry (DPV) Brdicka reaction

Electrochemical measurements were performed with 747 VA Stand instrument connected to 746 VA Trace Analyzer and 695 Autosampler (Metrohm, Switzerland), using a standard cell with three electrodes and cooled sample holder (4 °C). A hanging mercury drop electrode (HMDE) with a drop area of 0.4 mm^2^ was the working electrode. An Ag/AgCl/3M KCl electrode was the reference and glassy carbon electrode was auxiliary electrode. The supporting electrolyte (1 mM [Co(NH_3_)_6_]Cl_3_ and 1 M ammonium buffer; NH_3_(aq) and NH_4_Cl, pH 9.6) was changed after five measurements. The DPV parameters were as follows: initial potential of −0.7 V, end potential of −1.75 V, modulation time 0.057 s, time interval 0.2 s, step potential 2 mV, modulation amplitude -250 mV, E_ads_ = 0 V. All experiments were carried out at temperature 4 °C (Julabo F25, Germany). For smoothing and baseline correction the software GPES 4.9 supplied by EcoChemie was employed.

### 2.8 Statistical analysis

Data were processed using MICROSOFT EXCEL® (USA) and QCExpert software (TriloBite, Statistical Software, Czech Republic). Results are expressed as mean ± standard deviation (S.D.) unless noted otherwise. Differences with p < 0.05 were considered significant (t-test was applied for means comparison).

## 3. Results and Discussion

### 3.1 Influence of different sulphur forms on potato plants growth, yield of tuber and element content

**Figure 2.**
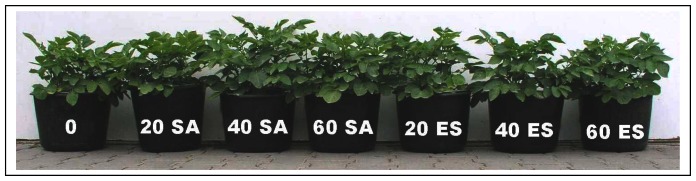
Pictures of plants exposed to ammonium sulphate (SA) and elemental sulphur (ES). Numeric data correspond to applied dosage in mg/kg introduced into the soil.

Tubers of Marabel cultivars were potted into soil with well-balanced nitrogen level and with various levels sulphur sulphates or elemental sulphur. All plants were growing without physiological and growth disorders. Sulphate application resulted in the moderate plant biomass enhancement, but no changes in dry mass were determined compared to control plants. On the other hand considerable differences in biomass production were determined at plants supplemented with elemental sulphur. These experimental plants were depressed with slightly reduced aerial parts in comparison with control plants (Fig. 2).

It clearly follows from the results obtained that content of nitrogen determined in aerial plant parts did not significantly change between experimental groups. Only plants treated with the highest dosage of elemental sulphur demonstrated significantly higher (p < 0.05) nitrogen content compared to other experimental groups. In addition the significant magnesium content increase was also determined in the plants cultivated in the presence of the highest dose of elemental sulphur compared to plants treated with the lower dosages. Moderate phosphorus content enhancement was also determined at experimental plants with application of elemental sulphur. The differences in potassium content were marked. These analyses showed that elemental sulphur application resulted in the considerable changes in levels of basic elements in plants. Supplementation the plants by ammonium sulphate (applied also directly into soil) led to the significant changes in calcium levels in plants. Particularly, under the highest sulphur concentration the calcium level in plants increased more than four times compared to control plants. Changes in sulphur levels were well evident especially. All experimental data are summarized in Tab. 2.

During the investigation of the influence of different sulphur forms on yield of potato tubers we did not observe significant changes. Average weight of tubers of control plants per one experimental pot was 355 g. Application of sulphur in both forms resulted in moderate potato tubers weight reduction per one experimental pot compared to control group; average value ranged from 320 to 350 g per one experimental pot.

Chemical analyses of elements (basic nutrients) in tubers demonstrated very low calcium and magnesium content; during whole experiment their content varied slightly. Similarly the phosphorus level was almost constant at all experimental groups. Nitrogen content was decreased at all experimental groups compared to control plants. On the other hand the sulphur contents were slightly increased in comparison with control plants. It clearly follows from the results obtained that sulphur application had negligible effect on monitored parameters. All data are summarized in Tab.3. In contrary with our results Bloem *et al.* reported that total sulphur content considerable enhanced after sulphur supplementation in the potassium sulphate form [25].

### 3.2 Effect of sulphur application on growth characteristics and cadmium uptake

McLaughlin *el al.* detected that despite of free Cd(II) ions reduction due to presence of SO_4_^2-^cadmium content in plant tissues was similar to control ones. In our experiments the influence of sulphur supplementation on cadmium(II) ions levels in potato plants was investigated. We determined that total cadmium content in fresh tuber mass after ammonium sulphate addition did not change (decrease of cadmium content about 1 % per 10 mg/kg of the applied ammonium sulphate). In the case of elemental sulphur total cadmium content in potato tubers was reduced about 5 % per 10 mg/kg of the applied elemental sulphur. Thanks to this fact it was possible to determine decrease in cadmium content in tubers of plants treated with the highest elemental sulphur dosage for more than 30 % in comparison with control plants. The significantly lowest cadmium content (p < 0.05) was determined in tissues of plants treated with the highest dosage of elemental sulphur (0.64 mg Cd/kg) compared to control plants (0.82 mg Cd/kg). Besides fresh mass cadmium content in dry mass of tubers was in close relation to cadmium content in fresh tuber mass. Cadmium content in both fresh tubers mass and dry tubers mass positively correlated with aerial parts weight (r = 0.793 and r = 0.824, respectively) and negatively correlated with the total sulphur content (r = -0.540 and r = -0.620, respectively).

The work reporting on significant enhancement of cadmium content in aerial plants parts of wheat in environment with increasing sulphate ions concentration was recently published. Increasing cadmium uptake by roots of the plants can be considered as the result of easier formation of complexes containing sulphur in their structures [27]. Cui *et al.* were interested in elemental sulphur influence on cadmium uptake [28-30]. Their model plants – maize – responded to elemental sulphur application by increasing of cadmium content in roots as well as aerial plant parts. Our experiments with potato plants (*Solanum tuberosum*) demonstrated moderate cadmium content increase in aerial plant parts after elemental sulphur application and more expressive cadmium content increase after sulphate application as 3 % per 10 mg/kg of the applied ammonium sulphate. After elemental sulphur application changes in cadmium content in aerial plant parts were negligible.

**Figure 3.**
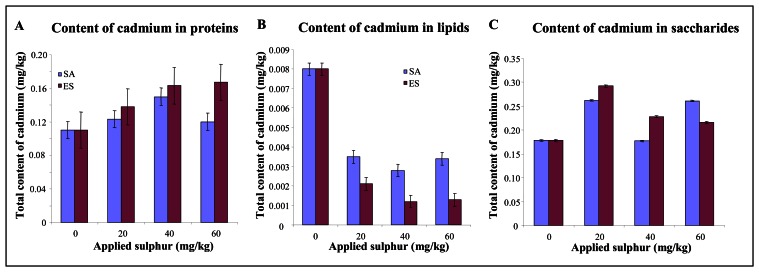
Cadmium content in (**A**) proteins, (**B**) lipids or (**C**) saccharides and ash obtained from potato plants treated with elemental sulphur and ammonium sulphate (0, 20, 40 and 60 mg/kg).

We also aimed our attention on the cadmium content in proteins. The content was slightly enhanced with increasing amount of applied sulphate as well as elemental sulphur. Cadmium content enhancement was about 5 % per 10 mg/kg of the applied sulphur form (Fig. 3). The changes observed can be related with the cadmium deposition into proteins, where the toxicity of these ions can be markedly lowered. The very interesting result was obtained in the case of cadmium content determination in lipid fraction. Application of sulphate as well as elemental sulphur resulted in significant cadmium content reduction in lipid fraction compared to control plants (Fig. 3). Moreover the content of cadmium determined in soluble carbohydrates and ash of plants treated with sulphates or elemental sulphur slightly enhanced compared to control plants.

### 3.3 Total thiols content in plants

**Figure 4.**
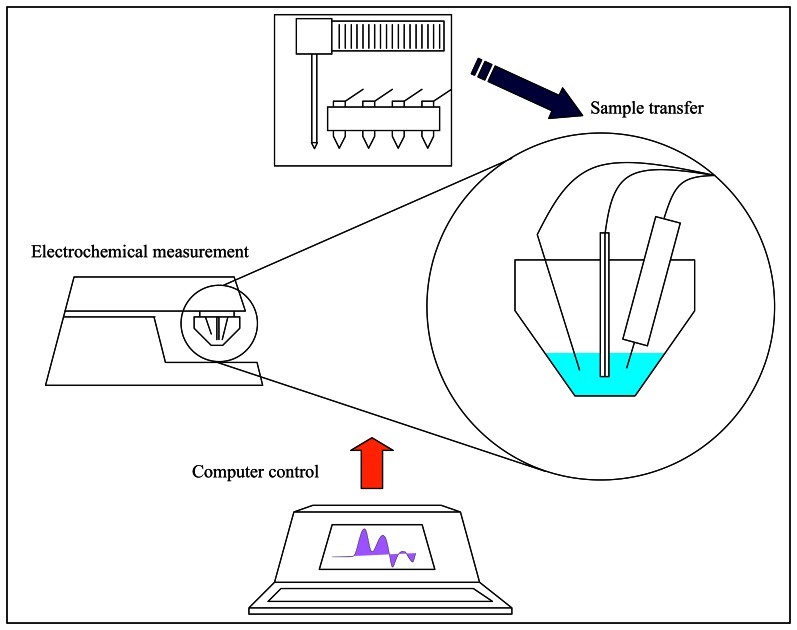
Scheme of automated electrochemical detection of thiols.

Peptides or proteins rich in sulphur aminoacids play many crucial roles in plants including participations in detoxification of heavy metals. In our several recently published papers we demonstrated the importance of these molecules in plat protection against heavy metals. To detect these stress peptides and proteins we employed electrochemical techniques [31-45]. One of the most sensitive electrochemical methods is differential pulse voltammetry Brdicka reaction. The main drawback of this method was non-automated analysis of samples. This obstacle can be overcome by automated electrochemical analyzer (Fig. 4).

**Figure 5.**
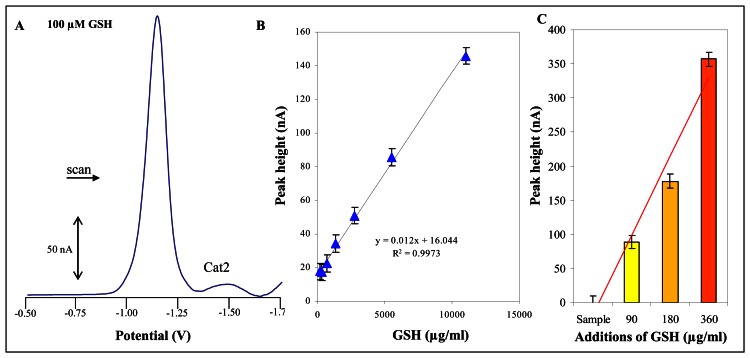
Electrochemical determination of glutathione by using of automated electrochemical analyzer (sample injection was 10 ml). (**A**) Typical voltammogram of 100 μM GSH. (**B**) Dependence of height of Cat2 signal on GSH concentration. (**C**) Height of Cat2 signal measured after standard GSH additions (90, 180 and 360 μg/ml).

In the extracts of plants prepared according to protocol mentioned in “Materials and Methods” section low molecular mass thiols such as cysteine, glutathione and phytochelatins can be found. We calibrated automatic analysers on detection of the most abundant low molecular mass thiol – reduced glutathione (GSH). This peptide gives well-developed catalytic signal Cat2. Typical DP voltammogram of 100 μM GSH is shown in Fig. 5A. The dependence of Cat2 signal height on GSH concentration was strictly linear with the equation of the straight line as y = 0.012x + 16.044, R^2^ = 0.9973 and relative standard deviation about 5.5 % (Fig. 5B). Thiols quantification (as GSH) was carried out by the standard addition method. The additions of GSH (90, 180 and 360 μg/ml) into extracts from potato plants are shown in Fig. 5C. Relative standard deviation additions measurement was about 10 % and was probably caused by the influence of biological matrix. The questions of low molecular mass thiols detection are still highly topical, but their quantification is relatively difficult due to their physico-chemical properties and interactions with other molecules in a biological sample.

**Figure 6.**
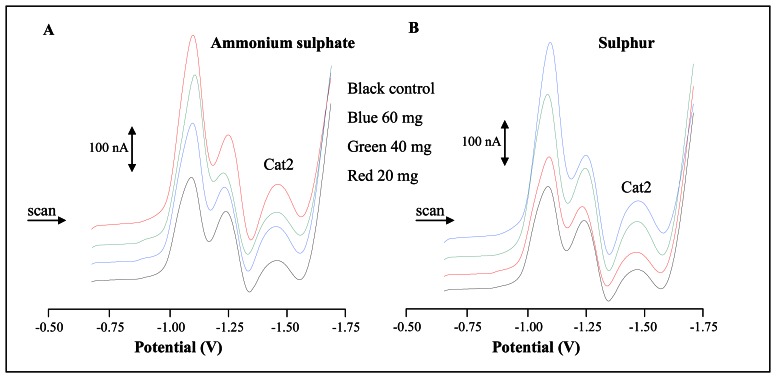
DP voltammograms of plants extracts after (**A**) ammonium sulphate and (**B**) elemental sulphur application.

**Figure 7.**
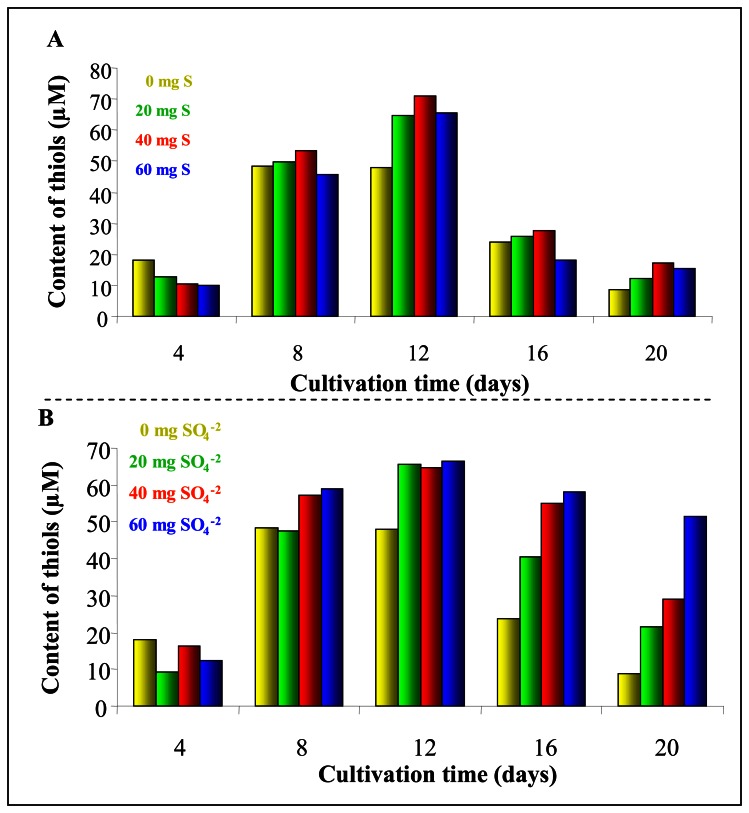
Content of low molecular mass thiols in potato cultivated in the presence of (**A**) ammonium sulphate or (**B**) elemental sulphur.

Typical DP voltammograms of extracts from plants cultivated in the presence of ammonium sulphate or elemental sulphur are shown in Figs. 6A,B, respectively. Biological extracts gave well distinguishable and typical electrochemical responses. Catalytic signal Cat2 was utilized for quantification of content of low molecular mass thiols. Character as well as behaviour of curves of plant extracts (exposed to ammonium sulphate and elemental sulphur) was similar. The content of low molecular thiols was lower at 4^th^ day of cultivation in the presence of sulphates or elemental sulphur compared to control (Fig. 7). Eight days after supplementation the highest thiols level was determined in extracts of plants treated with 20 and 40 mg/kg of elemental sulphur. After twelve days of the treatment enhancing of thiols level was observed in all experimental groups regardless to applied sulphur form and its concentration.

### 3.4 Sulphur supplementation influence on Phytophora infestans infection

We were interested in the issue if the foliar application of ORIN (commercial product containing elemental sulphur, doses and time of the cultivation was the same as in case of application of elemental sulphur or sulphates, Tab. 1) could influence the infection of potatoes by late blight. Bloem *et al.* and Mizuno *et al.* reported on the positive effect of elemental sulphur application to *Rhizoctonia solani* and *Streptomyces spp.* on infection development [26,46]. However sulphur application did not result in statistically lower frequency of infection and its development compared with control plants. On the contrary the highest applied concentration of ORIN resulted in the highest frequency of infection. This significant difference compared to control plants can be explained by the processes of elemental sulphur oxidation on the leaf surface as well as in the leaf mesophyll. During this process oxides of sulphur can damaged the protective barriers, such as cuticle or epidermis. Therefore this can lead to easier penetration of late blight. In addition sulphur oxides can entry to the leaves and may damage leave structures as well as interfere with physiological processes, such as photosynthesis. We can conclude that the higher content of elemental sulphur foliar application assists to late blight infection development.

**Figure 8.**
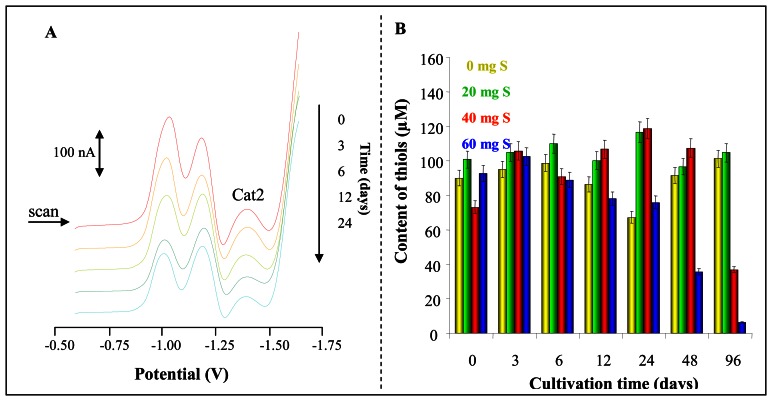
(**A**) Typical DP voltammograms of extract from potato plants exposed to *Phytophora infestans* infection. (**B**) Changes in thiols levels during infection development.

Moreover we determined content of thiols. Typical DP voltammograms of extracts of plants harvested at the beginning and at 3^rd^, 6^th^, 12^th^, and 24^th^ day of the experiment (Fig. 8A). The level of thiols did not change much between 0^th^ and 3^rd^ day of the cultivation. Their content during these days varied, but the differences were not significant. The most significant differences were evident after 96 hours long infection. We determined that total thiols content in plants supplemented with the lowest concentrations of elemental sulphur was similar to content of the substances in previous experimental days. Nevertheless decrease in thiols content in plants treated with the highest elemental sulphur concentration (40 and 60 mg/kg) was well evident (Fig. 8B).

## 4. Conclusion

Multiinstrumental point of view on specific experimental task should bring unique results. Here, we attempted to investigate the effects of supplementation of sulphur (as sulphates or elemental sulphur) on nutrients and thiols content. Moreover we described the ability of these plants to withstand treatment with toxic cadmium(II) ions and infection by late blight. It can be concluded that the supplementation by sulphur has considerable effect on plants and enhances protective mechanisms against heavy metals.

## Figures and Tables

**Table 1.** Scheme of pot experiment.

**Experimental group**	**Sulphur form used**	**Sulphur dosage (mg S/kg soil)**	**Way of application**
Control	Control	0	Without application
20 SA	(NH_4_)_2_SO_4_	20	Into soil
40 SA	(NH_4_)_2_SO_4_	40	Into soil
60 SA	(NH_4_)_2_SO_4_	60	Into soil
20 ES	Elemental	20	Into soil
40 ES	Elemental	40	Into soil
60 ES	Elemental	60	Into soil

**Table 2.** Content of nitrogen, phosphorus, potassium, calcium, magnesium and sulphur in aerial plant parts (herb).

Element	Statistical parameter	Control	Form of sulphur application					
			Ammonium sulphate			Elemental sulphur		
			20 SA	40 SA	60 SA	20 ES	40 ES	60 ES

Nitrogen	mean	1.2	1.1	1.1	1.0	1.0	1.1	1.1
	S.D.*	0.1	0.3	0.1	0.1	0.1	0.2	0.2

Phosphorus	mean	0.07	0.07	0.07	0.06	0.06	0.07	0.08
	S.D.*	0.02	0.03	0.01	0.01	0.01	0.02	0.02

Potassium	mean	5.5	5.6	5.8	5.6	5.0	4.6	4.8
	S.D.*	0.4	0.8	0.7	0.8	0.5	0.5	0.5

Calcium	mean	3.8	4.0	11	19	3.7	3.9	3.8
	S.D.*	0.7	0.1	1	15	0.5	0.1	0.4

Magnesium	mean	0.59	0.56	0.52	0.46	0.57	0.58	0.60
	S.D.*	0.08	0.05	0.09	0.06	0.06	0.03	0.04

Sulphur	mean	410	550	624	692	613	614	627
	S.D.*	1	1	3	1	1	1	1

* … standard deviation.

**Table 3.** Content of nitrogen, phosphorus, potassium, calcium, magnesium and sulphur in tubers.

Element	Statistical parameter	Control	Form of sulphur application					
			Ammonium sulphate			Elemental sulphur		
			20 SA	40 SA	60 SA	20 ES	40 ES	60 ES

Nitrogen	mean	1.9	1.8	1.7	1.6	1.7	1.6	1.8
	S.D.*	0.1	0.1	0.1	0.1	0.1	0.1	0.2

Phosphorus	mean	0.25	0.26	0.27	0.26	0.24	0.30	0.27
	S.D.*	0.01	0.01	0.01	0.01	0.01	0.06	0.01

Potassium	mean	2.9	2.8	2.9	2.9	2.8	2.8	2.9
	S.D.*	0.1	0.1	0.1	0.1	0.1	0.1	0.1

Calcium	mean	0.06	0.06	0.06	0.07	0.06	0.06	0.06
	S.D.*	0.01	0.01	0.01	0.02	0.01	0.01	0.01

Magnesium	mean	0.13	0.14	0.14	0.14	0.15	0.15	0.14
	S.D.*	0.01	0.01	0.01	0.01	0.01	0.01	0.01

Sulphur	mean	168	179	174	168	169	164	183
	S.D.*	1	1	1	2	1	1	1

* … standard deviation.
